# Comparing Machine Learning and Binary Thresholding Methods for Quantification of Callose Deposits in the Citrus Phloem

**DOI:** 10.3390/plants11050624

**Published:** 2022-02-25

**Authors:** Stacy Welker, Amit Levy

**Affiliations:** Citrus Research and Education Center, University of Florida, Lake Alfred, FL 33850, USA; stacy.welker@ufl.edu

**Keywords:** callose, phloem, supervised machine learning, bioimagery data collection, fiji, PlasmodesmataIlastik

## Abstract

Callose is a polysaccharide that can be fluorescently stained to study many developmental and immune functions in plants. High-throughput methods to accurately gather quantitative measurements of callose from confocal images are useful for many applications in plant biology. Previous callose quantification methods relied upon binary local thresholding, which had the disadvantage of not being able to differentiate callose in conditions with low contrast from background material. Here, a measurement approach that utilizes the Ilastik supervised machine learning imagery data collection software is described. The Ilastik software method provided superior efficiency for acquiring counts of callose deposits. We also determined the accuracy of these methods as compared to manual counts. We demonstrate that the automated software methods are both good predictors of manual counts, but that the Ilastik counts are significantly closer. Researchers can use this information to guide their choice of method to quantify callose in their work.

## 1. Introduction

Callose is a polysaccharide, composed of β-1,3-linked glucose, which is found throughout the plant body and performs a variety of structural, developmental, and immune functions [1,2,3]. Aniline blue is a fluorescent staining compound that binds to callose in plant tissue [4]. Because this stain is safe to handle and easy to use, fluorescent imagery of callose is often employed to study the biological processes in which it plays a role [5,6,7,8]. Increasingly, quantitative data from imagery is needed to further the understanding of the role of callose in plant molecular biology. Hundreds of callose deposits can be present in a single image of plant phloem tissue, so it can be extremely time-consuming to collect counts accurately without the aid of specialized software.

A method was previously published that details the automated quantification of callose using local binary thresholding in ImageJ [6]. Binary thresholding methods rely on the premise that the pixels of the object of interest contrast substantially with the pixels of the background of the image. Such methods are sensitive to noise, the presence of artifacts with the same brightness as the object to be measured, and user calibration of the various filter and threshold parameters [9]. Data can be lost when important objects are erroneously segmented to the background instead of the foreground.

In recent years, machine learning technology has greatly improved, and it has been applied to the problem of extracting quantitative data from images. However, the difficulty inherent in acquiring knowledge about image segmentation and machine learning can prevent biologists from being able to use it in their work [10]. A software program called Ilastik was developed that uses supervised machine learning to collect quantitative data from images [11]. Ilastik can use features other than brightness to threshold pixels, such as texture and scale, which can allow it to overcome the limitations of binary thresholding [12]. The expert user identifies the objects of interest and labels them for the Ilastik classifier. The software then uses a decision tree-based algorithm to predict the identity of objects in new images, based on the features of the objects in the user-labeled images. 

Other methods of automated quantification of callose in plant tissue images which also use machine learning technology have been published previously [5,13]. However, little effort has been made to quantitively compare callose formation counts obtained by expert human observers, traditional binary thresholding methods, and supervised machine learning methods. Computer-aided quantification of callose deposits can greatly accelerate exploration into the physiological processes in which callose plays a part, but it has not been empirically demonstrated that such methods provide results that approximate the counts that were manually acquired. Information is needed about the accuracy, so that researchers can confidently make decisions about which method is appropriate. 

In this work, our objective was to assess the sensitivity of the local binary thresholding method and supervised machine learning method in analyzing cellular fluorescent microscopy images. The counts produced by the automated methods were compared to manual counts of callose deposits in the same set of images of citrus tissue. Based on the previous work with each method, it was thought that supervised machine learning counts were closer to manual counts than those produced with local binary thresholding. Counts were collected from images of fluorescently stained phloem tissue, where an abundance of callose deposits were expected. Callose counts were also taken from stained pith tissue, where no callose deposits were expected, but fluorescent artifacts could be present. As a control for the stain, images were also taken of unstained phloem tissue. Our results indicate that supervised machine learning exhibited greater sensitivity as compared to binary thresholding. These findings provide a test case for employing machine learning for phloem callose measurements and can provide support for similar analysis in microscopic images of other cellular components and plant tissues. 

## 2. Results

### 2.1. Supervised Machine Learning Was Identified as More Sensitive in Detecting Phloem Callose

**Figure 1:**
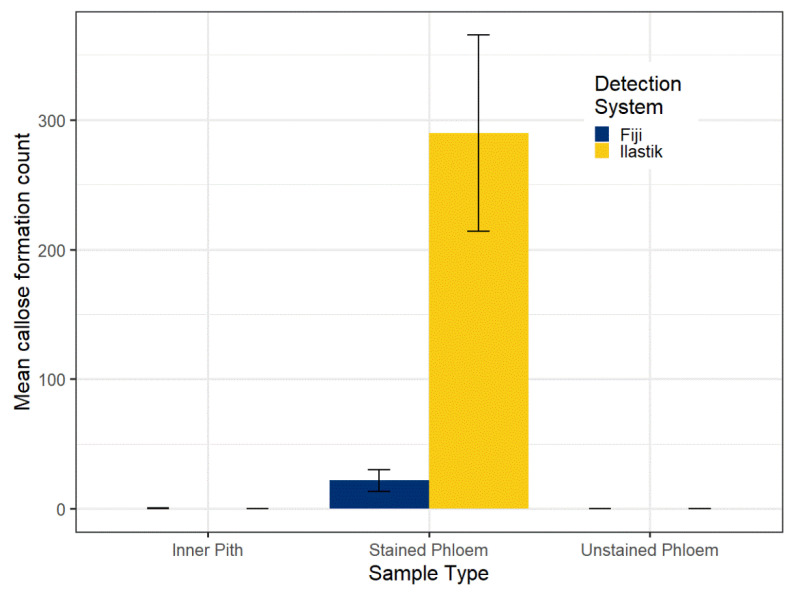
Mean count of callose formations (per 10× field) in citrus phloem and pith tissue. Callose formation counts were obtained from confocal images using either Ilastik, which uses a supervised machine learning method, or Fiji, which applied a local binary threshold method. No callose formations were detected in the unstained tissue or stained inner pith using either method. Error bars represent standard error, n (stained phloem) = 15, n (unstained phloem) = 15 n (inner pith) = 9. Contrast between the number of formations detected using Ilastik or Fiji in the phloem is significant at *p =* 0.001, according to Wilcoxon signed-rank test results.

Phloem and pith tissue from three-year-old citrus was collected and stained with aniline blue. Images were collected with the confocal microscope. After feature reduction with a mean filter, a machine learning algorithm was trained using the Ilastik software. This algorithm was able to differentiate between callose, non-fluorescing background, and fluorescent artifacts in the images. Counts of callose were obtained from the same images manually and by using a Fiji local binary thresholding method which segmented the callose deposits from the background. There were significant differences between the number of callose deposits detected by each testing system in the phloem (Figure 1). In the stained phloem tissue, the mean number of deposits (per 10× field) detected with Fiji was 21.9 (standard error = 8.5), while with Ilastik, the mean count (per 10× field) was 289.5 (standard error = 75.7). As controls, we used unstained phloem tissue and stained inner pith tissue, where no callose should be present. No fluorescent objects were detected in the unstained phloem tissue or stained inner pith. 

### 2.2. Binary Thresholding Counts Were Significantly Different from Manual Counts, but Supervised Machine Learning Counts Were Not

**Figure 2:**
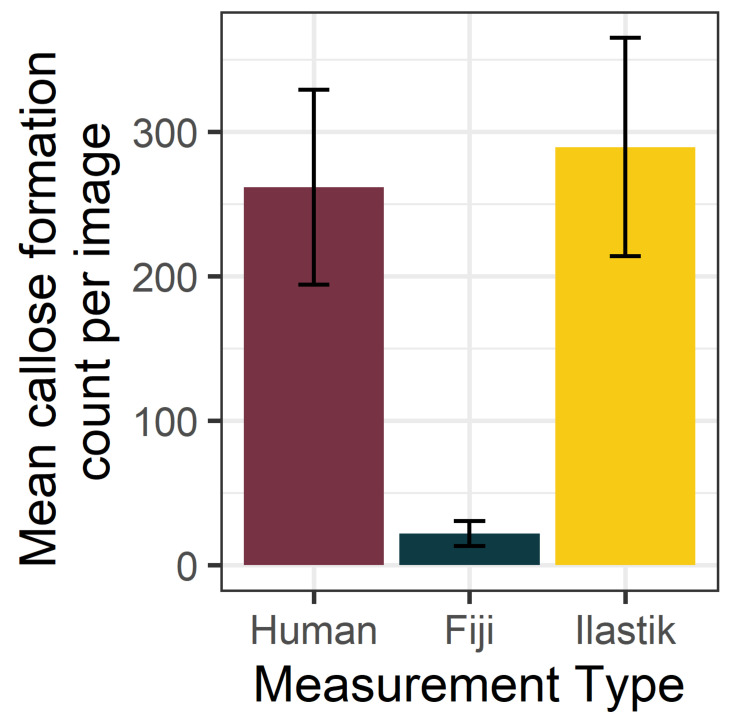
Mean counts of callose formations (per 10× field image) in citrus phloem tissue. Callose formation counts were obtained from the same set of confocal images, using the Ilastik supervised machine learning method, the Fiji local binary threshold method, and a human counter. Error bars represent standard error, n = 15. The contrast between Fiji counts and manual counts is significant, at *p ≤* 0.001, according to Friedman test results.

Phloem and pith tissue from three-year-old citrus was collected and stained with aniline blue. Images were collected with the confocal microscope. Manual counts of callose deposits were collected from each image. After feature reduction with a mean filter, counts were collected with the Ilastik software and Fiji method. Figure 2 illustrates the mean and standard error of the counts obtained from the stained phloem images by each detection method. According to the Friedman test results, there were significant differences between the counts with χ^2^(2) = 21.6, *p* < 0.001, and an effect size of 0.72. Post hoc analysis was performed using pairwise Wilcoxon signed-rank tests, with a Bonferroni correction for multiple comparisons (Table 1). The counts produced by Fiji were significantly different from manual counts, but Ilastik counts were not. The Ilastik counts were higher than the human counts, but the difference was not statistically significant.

### 2.3. Callose Was Correctly Identified by Both Software Methods in Pith Outer Layers

**Figure 3:**
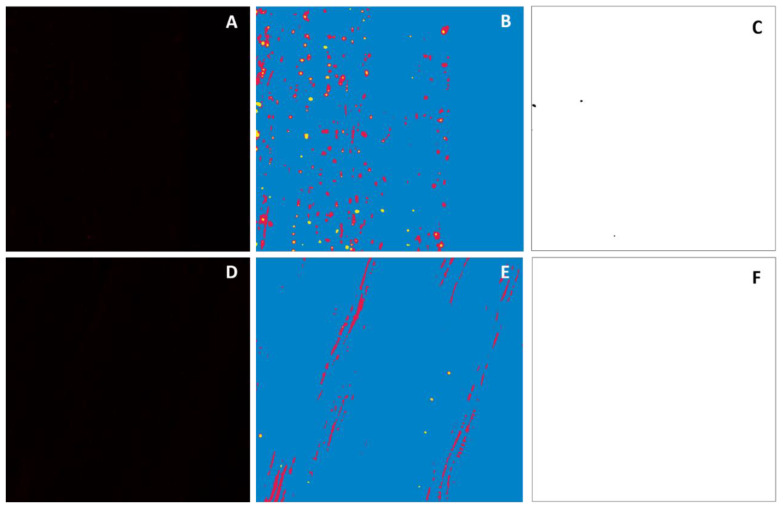
Comparison between raw confocal images, segmentation results from Ilastik, and threshold results from Fiji. (**A**–**C**): A raw confocal image, Ilastik segmentation results, and Fiji threshold results, respectively, of callose deposits in citrus stem phloem tissue. (**D**–**F**): A raw confocal image, Ilastik segmentation results, and Fiji threshold results, respectively, of callose deposits in citrus stem outer pith tissue. (**B**,**E**): Blue represents non-fluorescent background. Red represents fluorescent artifact material, such as epidermis. Yellow represents fluorescent callose deposits. (**C**,**F**): White represents non-callose background, and black represents callose deposits.

Callose deposits were unexpectedly detected in the outer pith tissue images with Fiji (mean = 0.27, standard error = 0.15) and Ilastik (mean = 68.4, standard error = 15.1). The pith images were manually reviewed and found to contain callose deposits, as reported by the software. The segmented images produced by Ilastik revealed the differences in artifact topography between the phloem and pith tissue (Figure 3). Based on the image topography, it appeared that the identified formations were callose deposits that clung to the outer pith as the phloem was peeled away. No objects were detected in the inner pith samples (Figure 1), and this was confirmed by visual inspection, which revealed a lack of fluorescence in the images. The results confirmed that callose deposits from the phloem tissue adhere to the outer pith layer during the tissue acquisition process

### 2.4. Counts from the Supervised Machine Learning Method Are a Better Approximation of Manual Counts

Negative binomial regressions were performed to assess which software method was a better predictor of manual counts of callose deposits in the citrus phloem tissue. Table 2 contains the results of the regression between manual counts and the counts obtained with the Ilastik method. Table 3 contains the results of the regression between manual counts and the counts obtained with the Fiji method. Both software methods are significant predictors of manual counts. Three measures of fitness were computed for each model (Table 4). The Ilastik count was model showed less uncertainty and information loss and had a better fit to the manual counts. 

## 3. Discussion

The results indicate that both software methods can be used as a substitute for manual counts in experiments where callose is used as a physiological indicator in plants. Statistical analysis of the counts produced with data from the two image segmentation methods reveals that Ilastik counts resembled human counts more closely than those from Fiji. Fiji counts are a significant predictor of manual counts, but they differ from the manual results significantly. Ilastik counts were a better predictor of manual counts, according to three measures of model fitness. However, Ilastik reported a higher mean count than the human observer. The trained classifier was likely over-generalizing and including some fluorescent artifacts in the callose category, but this difference was not significant.

Both software methods identified callose deposits in outer pith tissue. This raised the possibility of a lack of specificity, because callose deposits are normally a feature found the phloem, not the pith. Upon manual review, the human counter also found callose deposits in the outer pith tissue. When aniline blue-stained images from the inner and outer piths were obtained and run through the Ilastik classifier, no fluorescence could be detected in the inner pith. This suggests that some phloem tissue may cling to the outer pith after the peeling process is complete, leaving detectable callose deposits. Because the classifier had been trained to recognize callose deposits in the phloem, it may not be able to differentiate between artifacts and callose in the context of the pith tissue. The appearance of callose where none was expected required further experimentation to confirm the identity of the objects that were detected by the classifier. This underscores the importance of re-training the Ilastik classifier for each new tissue type to ensure maximum accuracy and efficiency.

The analyses shown here indicate that the Ilastik segmentation method will be more suitable for images where there is low contrast between the desirable objects and background, or where multiple similar targets need to be quantified. The Ilastik classifier appears to be more sensitive in detecting callose deposits than the binary thresholding method, and acceptable specificity is obtained even when fluorescent artifacts are present in images and have a similar brightness level to the callose deposits. Fewer pre-processing steps are needed to obtain satisfactory results, but the accuracy of the identification was improved by applying a mean filter to the images with Fiji before applying the classifier. Feature reduction is a common step in machine learning-based data collection processes [14]. In this application, it appears necessary to reduce the complexity of the images in order to make it less likely that noise artifacts will be mistaken for features by the software. 

One limitation of this study is the fact that the human counter had previous experience with both types of software counting methods. This may have produced some bias in the counts. Ideally, the bias would be reduced by obtaining counts of the same images by multiple human observers who were unaware of the capabilities of each software. However, multiple manual counts were not feasible for this project. Because counting callose deposits in the citrus phloem is a relatively simple task, it may be more difficult to obtain highly accurate counts from a trained machine learning classifier with more complex types of images. Researchers should evaluate different counting methods and choose the most appropriate one for their datasets and resources. In any imagery data collection project, care must be taken to adjust settings and feature reduction until they match the human observation as much as possible for all software methods. 

## 4. Materials and Methods

### 4.1. Plant Material and Tissue Collection

The plants used in this study were healthy ‘Valencia’ sweet orange trees (Citrus sinensis L. Osbeck), which were approximately 3 years old. The plants were kept in an air-conditioned greenhouse in Lake Alfred, Florida, with an ambient temperature of 27 °C. The trees were grown in 1-gallon pots, with water applied 2 to 3 times weekly, as needed. The water contained 20–10–20 fertilizer at 15% concentration. Samples for the initial classification were collected in December 2020. Tissue samples were obtained from the branch portion of the mature flush of the trees, approximately 7–10 cm from the leaves. 

For the initial comparison between Fiji and Ilastik, two types of samples were collected: bark samples that contained phloem tissue and pith samples that were largely free from phloem tissue. A scalpel was used to peel away a section of bark from the branch, with the phloem tissue adherent to the inside. The dimensions of the bark peels were approximately 0.5 to 1 cm wide and 2 to 3 cm in length. A scalpel was also used to peel away portions of the stem pith with similar dimensions to the phloem samples. In total, three samples were collected each from three trees: one phloem sample to be stained with aniline blue, one phloem sample to remain unstained, and one pith sample that was also stained with aniline blue. All samples were plunged immediately after collection into 1.5 mL Eppendorf tubes filled with 85% ethanol. After the ethanol fixation, the phloem samples were divided into a group that was stained and a group that was not stained.

After the initial comparison analysis was completed, more tissue samples were collected and stained to further examine callose which was identified in the pith images from the previous samples. In November 2021, one sample each was collected from three healthy 2-year-old ‘Valencia’ sweet orange trees that were kept in the same location and conditions as the initial experiment. Stem segments that were 3 cm in length were cut from mature flush branches, approximately 10 cm from the leaves. The stem segments were placed immediately in Eppendorf tubes filled with 85% ethanol. Each stem segment was kept submerged in the ethanol for 24 h to fix and destain the tissue. The stem segments were removed from the ethanol and dissected with a scalpel into the phloem, outer pith, and inner pith layers. 

### 4.2. Tissue Staining

All samples were transferred from the ethanol solution to a 0.01% Tween-20 solution. The unstained samples remained in this solution until images were taken, for approximately 2 h. After rehydrating in the tween solution for 1 h, the other phloem and pith samples were placed in the stain solution. The stain consisted of a 0.6 M glycine solution, which contained 0.01% *w/v* aniline blue. The pH of the stain was adjusted to 9.5 with several drops of 5 M KOH from a transfer pipette. The samples were allowed to absorb the stain for 1 h. After staining, these samples were also transferred to the Tween solution until the images were taken.

After dissection, the samples that were collected to further examine the pith callose were treated with the same staining procedure as described above.

### 4.3. Image Collection

Five images were taken from each tissue peel, which resulted in a total of 15 images each for the three groups (stained phloem, unstained phloem, and stained xylem). A Leica SP8 confocal microscope was used to collect images, with the 10×/0.32 NA objective. The pinhole diameter was set at 330.5 µm. A 405 nm diode laser was used for excitation at 25% power, and a 475–525 nm band-pass filter was used for emission detection, with gain at 400.9 and offset at −0.09. The Leica Application Suite X software was used to convert the images to TIF format for data collection. The images produced in this step were used as the test set. Three citrus phloem images from a previous callose measuring experiment, stained with the same procedure and imaged using the same settings, were used as the training set [15].

For the pith callose samples, three pictures were collected from each type of tissue from three stem segments, making a sample of nine images for each tissue type (phloem, inner pith, and outer pith). These images were collected using the same microscope and settings as described above. For the outer pith samples, the morphology of each sample was examined, and images were taken only of the surface that contacted the phloem.

### 4.4. Manual Counts

All images of the first collection of phloem tissue were also manually counted by a human observer who was highly familiar with the appearance of callose deposits. The images were viewed at 200% magnification in Microsoft Paint. The observer counted one field of view at a time using a digital click counter. After each field of view was counted, the observer scrolled to the next field. The fields of view were counted in a serpentine pattern, starting at the top right and ending at the bottom left. If callose deposits were located at the bottom and right edges of a field, they were counted and used as a guide to scroll to the next field of view. Each callose deposit was marked with a bright dot to ensure it was counted. Each image was examined again at full magnification to make sure all callose deposits were counted. Counts were recorded for statistical analysis in a CSV file.

### 4.5. Image Pre-Processing for Ilastik

**Figure 4:**
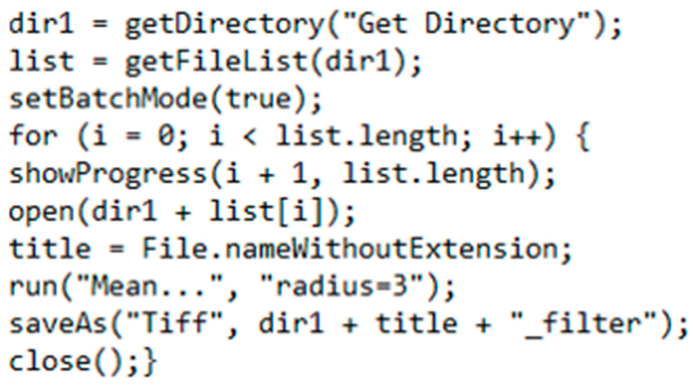
Text of a Fiji macro that was used to apply a filter to the images before processing with Ilastik. The macro gives the user a prompt to select a directory. It applies a mean filter, with a radius of three pixels, to all images in the directory. The filtered images are saved as a new file with “_filter” at the end of the name. A status bar allows the user to monitor progress and spot errors if they occur.

To reduce the effects of noise artifacts on the training of the algorithm, a copy of each image was pre-processed, with a mean filter and radius of three pixels using Fiji [16]. A macro was scripted to apply the filter automatically to all images in one session (Figure 4). The new filtered images were saved in a separate drive, leaving the originals unaltered. The same filter was applied to the pith images.

### 4.6. Supervised Machine Learning-Based Image Segmentation

**Figure 5:**
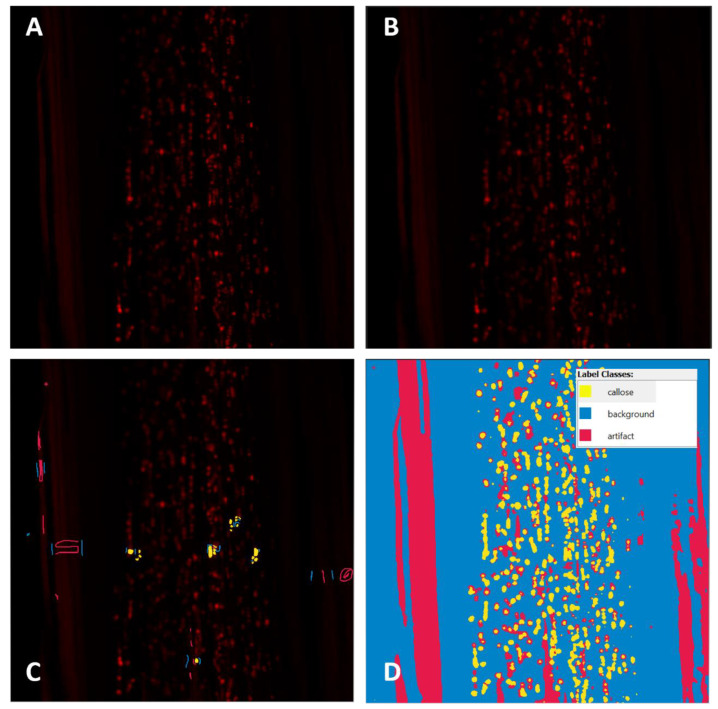
A stepwise illustration of the callose measurement process in citrus phloem using Ilastik. (**A**). An unaltered image of a high-callose section of citrus phloem, stained with aniline blue. (**B**). The same image after a mean filter with radius = 3 is applied with Fiji. (**C**). Three labels are chosen for the callose formations, stained cuticle artifact, and background. Several labels for each class are added initially; then, additional labels are added in areas of high uncertainty, until the algorithm produces accurate predictions in the whole training image. (**D**). A fully segmented image, with all objects classified correctly, according to the expertise of the human trainer.

Identification and measurement of callose formations was performed using a two-step workflow in the Ilastik software [11] (Figure 5). The first step was pixel classification. The three images, from a prior callose-measuring experiment, were loaded into the data input menu and used to train the algorithm [15]. The tissue in these training images had been stained, and images were taken with the same procedures outlined in the preceding methods sections. The images were chosen to each represent tissue with typical high, medium, or low-callose quantities. After the training images were loaded, the pixel features and feature size (sigma) were selected. Because computation time was not expected to become problematic, all features on all sigma scales were selected. This allowed pixels to be classified based on color, color intensity, resemblance to an edge, and texture. Next, the supervised training was performed. In this step, a separate color label was chosen to identify callose, background, or epidermis/cuticle artifact. Three examples of each object were labeled with the appropriately sized brush; then, the classifier was allowed to update to observe the results. Using the uncertainty overlay, areas of high uncertainty were labeled iteratively until the prediction layer showed satisfactory identification of the pixels. This process was repeated for all three images. The trained classifier was then run on all images in the test dataset, and the pixel classification data was saved.

In the second step, the object classification workflow was used to label regions of the images as object types (callose, background, or cuticle artifact), based on how pixels were classified. The raw training images, in addition to the associated probability maps, which were obtained in the pixel classification step, were loaded into the workflow. All standard features were selected to identify the objects. The neighborhood size was left at 30 × 30 pixels. The training process was similar to that in the pixel classification workflow. Three different labels were chosen for callose, background, and cuticle artifact objects. Three of each type of object were labeled; then, further labels were placed on objects with highly uncertain identities, until all objects in the images were correctly identified, according to the trainer’s expertise. When the training process was complete, the classifier was applied to the images in the test dataset. The data collected from every object in each image was exported as CSV files to be used for statistical analysis. 

The same workflow was used to collect counts of callose formations and artifacts from the images for the pith callose examination.

### 4.7. Local Binary Thresholding and Segmentation

To create a comparison dataset which was acquired using a binary segmentation method, the initial set of test images that were classified with Ilastik were processed with a workflow in Fiji, which was described previously [6,15].

### 4.8. Statistical Analysis

All statistical analysis was performed using Ref. [17]. The CSV files that contained information about objects in the images were combined and cleaned, such that the measurements were grouped by the tissue type (stained phloem, unstained phloem, or pith). The count of callose objects per image was obtained from the CSV files, and summary statistics were computed for each count dataset. Because no callose objects were detected from the unstained phloem tissue, only the stained phloem and pith were included in statistical tests.

Linear regression models were examined to compare the Fiji and Ilastik counts as predictors of human counts. This analysis was performed in other biological studies that examined machine learning count accuracy versus human-performed counts [18]. However, when the residuals of the linear models were tested for this citrus phloem study, they did not meet the assumptions of linearity and homogeneity of variance. Poisson, negative binomial, and hurdle models were considered because they are most appropriate for biological count data [19]. The negative binomial regression was chosen for both the Fiji and Ilastik counts because the variance was greater than the mean for all sets of counts. A hurdle model may also have been appropriate for the Fiji counts, but the negative binomial model was chosen so that both models could be compared. K–L R^2^ is the most appropriate measure for finding the proportion of variance explained by each regressor in nonlinear regression models, and this was found for both regression models with the {performance} package in Refs. [20,21]. RMSE was calculated for each model with the {Metrics} package [22]. Because the count datasets did not meet the assumptions for the parametric tests, such as ANOVA, the Friedman test for paired data was chosen to find differences in the mean counts, based on the type of measurement system [23]. The effect size for the Friedman test was found with the {rstatix} package [24]. Post hoc analysis was performed using pairwise Wilcoxon signed-rank tests with a Bonferroni correction for multiple comparisons. A Wilcoxon signed-rank test was also used to compare the callose deposit counts obtained by both software methods in the citrus phloem tissue.

## 5. Conclusions

The counts of callose deposits that were produced by the Ilastik supervised machine learning software were a good predictor of manual counts from the same images, although Ilastik may have counted some artifacts as callose. The Fiji counts from the same images were also significant predictors of human counts, but the counts differed significantly. The Ilastik method, described here, may allow significant biological differences in callose production to be easily detected in low contrast images, while saving time, compared to manual counts. Regardless of the automated method used, researchers should carefully evaluate the data produced by the software and ensure that the level of accuracy is appropriate for their objectives.

## Figures and Tables

**Table 1:** Post hoc comparison of count results using a Wilcoxon signed-rank test for paired samples. *p*-values were adjusted for multiple comparisons using the Bonferroni method (α = 0.05). Pair group size was 15 for all groups. Counts were obtained from the same set of fluorescently stained citrus phloem tissue manually, with Fiji, or with Ilastik. Asteriks indicate significant differences at *p* < 0.05.

Count Pair	Z (Test Statistic)	*p*-Value
Human–Fiji	120	<0.001 *
Human–Ilastik	35.5	0.52
Fiji–Ilastik	0	0.003 *

**Table 2:** Results of a negative binomial regression which evaluates the Ilastik counts of callose deposits as a predictor of manual counts from the same images ^1^. The counts were collected using each method from images of fluorescently stained citrus phloem tissue (at 10× magnification). Asterisks indicate significant differences at *p* ≤ 0.05.

Effect	Estimate	Standard Error	z-Value	*p*-Value
Intercept	3.86	0.24	16.22	<0.001 *
Ilastik count	0.004	<0.001	7.023	<0.001 *

^1^ Degrees of freedom = 14.

**Table 3:** Results of negative binomial regression which evaluates counts of callose deposits, obtained with Fiji, as a predictor of manual counts from the same images ^1^. Counts were collected using each method from images of fluorescently stained citrus phloem tissue (at 10× magnification).

Effect	Estimate	Standard Error	z-Value	*p*-Value
Intercept	4.75	0.28	16.75	<0.001 *
Fiji count	0.02	0.007	3.32	<0.001 *

^1^ Degrees of freedom = 14. * Indicate significant differences at *p* < 0.05.

**Table 4:** Comparison of model fitness between two negative binomial regressions, which assessed the counts obtained either by the Fiji or Ilastik method, as predictors of manual counts. Counts of callose deposits were from the same images of fluorescently stained citrus phloem tissue (at 10× magnification). Kullback–Leibler R^2^ (K–L R^2^) measures the proportion of uncertainty explained by the inclusion of a regressor in a general linear model. Root mean square error (RMSE) is a measure of model accuracy, where a lower number indicates a better fit between the predicted and observed data. Akaike information criterion (AIC) is an estimation of how much information is lost when a model is fitted to a dataset.

Predictor	K–L R^2^	RMSE	AIC
Ilastik	0.68	60.87	182.29
Fiji	0.34	329.26	194.18

## Data Availability

All imagery, data, and the R script used for analysis can be obtained through the corresponding author, Amit Levy (amitlevy@ufl.edu).
